# 

**Published:** 1882-12

**Authors:** 


### CONTENTS:

ART. I.—Nervous Force. Its Origin and Physiology.
By W. C. Barrett, M. D., D. D S. .......... 337
ART. II.—The Study of Diseases of Children. By W. B. Atkinson, M. D. .......... 354
ART. III.—Professional Etiquette .......... 369

### EDITORIAL, ETC.

Dental Colleges .......... 372

### BIBLIOGRAPHICAL NOTICES.

Colemans Dental Surgery and Pathology, Revised by Thos. Stellwager
M. D., D. D, S .......... 374
Dental Metallurgy. By Chas. J. Essig, M. D., D. D. S .......... 375
Quiz Compends. No. 1. Anatomy. By Samuel O. Potter, M. A., M. D .......... 375
Physicians Visiting List for 1883. P. Blackiston & Son .......... 375
The Independent Practitioner .......... 376

### MONTHLY SUMMARY.

The Speed of Thought .......... 376
The Use of Iodiform in Dental Practice .......... 377
Excision of Superior Maxillary and Inferior Dental Nerves .......... 378
Tetanus from a Carious Tooth .......... 379
Therapeutics and Physiology .......... 380
Changes in the Secretion of Milk under Influence of Medicine .......... 380
The Teeth in Diabetes .......... 381
Preparations of Aconite .......... 382
Lancing Gums of Children .......... 383
Toothless Persons .......... 383
Varnish Your Cavities .......... 383
To Gild without a Battery .......... 384
Excision of the Tongue .......... 384
Swallowing Artificial Teeth .......... 384

